# Clinical leadership and integrated primary care: A systematic literature review

**DOI:** 10.1080/13814788.2018.1515907

**Published:** 2018-11-26

**Authors:** Minke S. Nieuwboer, Rob van der Sande, Marjolein A. van der Marck, Marcel G. M. Olde Rikkert, Marieke Perry

**Affiliations:** a Department of Geriatric Medicine, Radboud University Medical Center, Radboud UMC Alzheimer Centre, Nijmegen, The Netherlands;; b Department of Geriatric Medicine, Radboud University Medical Center, Donders Institute for Brain Cognition and Behaviour, Nijmegen, The Netherlands;; c Radboud University Medical Center, Department of Geriatric Medicine, Radboud Institute for Health Sciences, Nijmegen, The Netherlands;; d Department of Primary and Community Care, Radboud University Medical Center, Radboud Institute for Health Sciences, Nijmegen, The Netherlands;; e Faculty of Health, Behaviour and Society, HAN University of Applied Sciences, Nijmegen, The Netherlands

**Keywords:** General practice/family medicine, general, integrated care, systematic reviews and meta-analyses, skills training

## Abstract

**Background:** Leaders are needed to address healthcare changes essential for implementation of integrated primary care. What kind of leadership this needs, which professionals should fulfil this role and how these leaders can be supported remains unclear.

**Objectives:** To review the literature on the effectiveness of programmes to support leadership, the relationship between clinical leadership and integrated primary care, and important leadership skills for integrated primary care practice.

**Methods:** We systematically searched PubMed, CINAHL, Embase, PsycINFO until June 2018 for empirical studies situated in an integrated primarycare setting, regarding clinical leadership, leadership skills, support programmes and integrated-care models. Two researchers independently selected relevant studies and critically appraised studies on methodological quality, summarized data and mapped qualitative data on leadership skills.

**Results:** Of the 3207 articles identified, 56 were selected based on abstract and title, from which 20 met the inclusion criteria. Selected papers were of mediocre quality. Two non-controlled studies suggested that leadership support programmes helped prepare and guide leaders and positively contributed to implementation of integrated primary care. There was little support that leaders positively influence implementation of integrated care. Leaders’ relational and organizational skills as well as process-management and change-management skills were considered important to improve care integration. Physicians seemed to be the most adequate leaders.

**Conclusion:** Good quality research on clinical leadership in integrated primary care is scarce. More profound knowledge is needed about leadership skills, required for integrated-care implementation, and leadership support aimed at developing these skills.

KEYMESSAGES

- Research to build a stronger evidence base for leadership and supportive leadership interventions is urgently needed to warrant the current emphasis on leadership in integrated primary care.
- Evidence on essential leadership skills adds that physicians require relational and organizational skills, as well as process-management and change-management skills.

## Introduction

**Figure 1.**
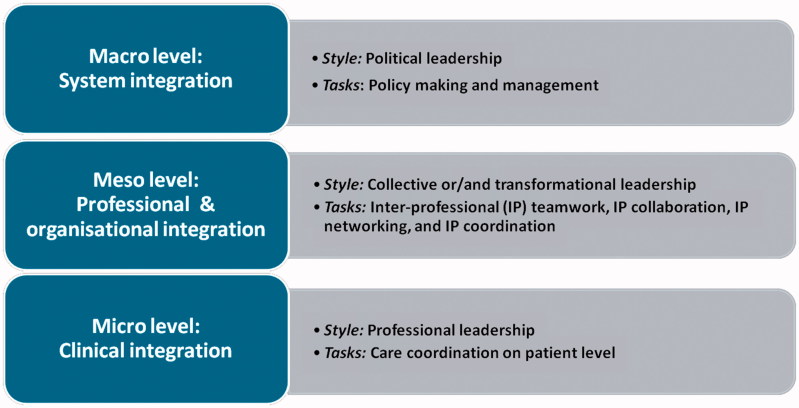
The three different levels of care integration and their leadership styles and tasks.

As numbers of chronically ill patients with complex healthcare needs are increasing, primary care professionals will be challenged to deliver integrated care. Integrated care is about ‘delivering seamless care for patients with complex long-term problems cutting across multiple services, providers and settings’ ([1], p. 58). It covers care processes that take place on the micro (clinical integration), meso- (professional and organizational integration) and macro (system integration) level (Figure 1) [2], and requires interprofessional care including teamwork, collaboration, coordination and networking [3]. Consequently, implementation of integrated care is a complex and sometimes even chaotic process, requiring a fundamental redesign of usual primary care [4,5].

Leadership is considered a prerequisite for integrated primary care [6–9] to give direction and align within organizations and interprofessional teams [10,11]. Worldwide, physician leadership is endorsed to foster collaboration with colleagues interprofessionally [9,12]. Therefore, physician leadership should exceed leading multidisciplinary meetings. It is also about the ability to change the care process, e.g. defining new roles for different professionals, handling different interests and implementing patient care coordination.

A review of studies in the hospital setting recently showed that nursing leadership might lead to higher patient satisfaction, lower patient mortality, fewer medication errors and fewer hospital-acquired infections [13]. Within the Chronic Care Model, the most accepted integrated-care model, leadership is recommended to enlarge the effectiveness of integrated care [14]. However, lack of leadership power is often reported in integrated-care studies [7,8] and few studies support the assertion that leadership advances integrated care [15].

Because of the diversity in autonomous professionals and the differences in care arrangements, experiences and views of professionals in primary care [16], it is plausible that leadership aimed at primary care integration requires specific leadership styles and skills (See Box 1 and Figure 1 for leadership styles and tasks) [17].

BOX 1 Leadership styles related to integrated care

Two important leadership styles can be distinguished in relation to integrated care:

- collective leadership (e.g. shared, collaborative, dispersed, distributed or team leadership) that involves the collective influence of team members and is based on social interactions [18].
- transformational leadership, a more hierarchical style, where leaders transform their followers by charisma and motivate them to achieve more than what is expected and challenge them to look beyond self-interest [19].

A recent scoping review identified collective leadership as the most important style to facilitate interprofessional care, although it remained unclear how this style was applied. Only a few studies described leadership skills needed for collaboration with colleagues with different professional or organizational backgrounds [20].

Several preparation and support programmes exist to develop leadership skills among healthcare professionals [20]. Most of these programmes target physicians and nurses (clinical leadership) in hospital settings [15], and only few address care integration [21]. Despite the broadly shared idea that leadership is essential for the delivery of integrated care, the nature and strength of the association between leadership and integrated primary care practice remains unclear [20]. In a review of the literature, we therefore, aimed to primarily study the effectiveness of leadership preparation and support programmes on integrated primary care practice. Furthermore, we explored the association between clinical leadership and integrated primary care practice and outcomes and skills required for effective clinical leadership in an integrated primary care context.

## Methods

### Search strategy

We performed a systematic review according to the PRISMA recommendations [22] (Prospero CRD42016036746). We searched the electronic databases of PubMed, CINAHL, Embase, PsycINFO up to 30 June 2018, including relevant synonyms for (1) Leadership AND (2) Integrated Care, namely ‘Chronic Care Model,’ ‘coordinated healthcare,’ ‘integrated health service,’ ‘collaborative healthcare,’ ‘interprofessional collaboration,’ ‘interprofessional cooperation,’ ‘inter organizational collaboration’ and ‘inter organizational cooperation,’ without restrictions regarding language or year of publication. Additionally, we performed the snowball method and manually searched systematic reviews on implementation of integrated care (Supplemental Material, available online).

### Inclusion criteria

For inclusion, articles had to (1) describe empirical research with quantitative and/or qualitative data collection, full text available; (2) address clinical leadership in an integrated primary care setting or collaboration between primary and hospital care; (3) focus on the effectiveness of leadership support and training, on required leadership skills and/or the association between leadership and integrated primary care practice; and (4) focus on the meso-level of integrated care (Figure 1).

Excluded were reviews, opinion papers, papers on health policy, papers solely situated within the hospital setting, and papers that report on clinical interventions with the focus on process indicators. We excluded studies on integrated care defined as public health programmes, oral health, telehealth, disease management, care pathways, educational programmes, and studies with the following perspectives: non-clinical leadership (management, governance, political, church, military, civic and lay leaders) and care integration not exceeding the micro level (care coordination).

### Selection of papers, critical appraisal and data extraction

After exclusion of duplicates, a first selection was made based on article titles by one reviewer (MN); then, abstracts were independently screened by two researchers (MP, MN). The relevant articles were read full-text and assessed for inclusion. In case of disagreement, discussion led to consensus or a third researcher was consulted (MvdM). To determine the level of agreement, Cohen’s к was calculated.

Subsequently, the studies included were appraised independently on methodological quality by two researchers (MP, MN). We used the mixed methods appraisal tool (MMAT) as this tool allows concomitant appraisal of qualitative, quantitative and mixed methods studies [23]. MMAT scores represent the number of criteria met, divided by four and translated in percentages; scoring varies from 25% (noted as *, low quality) to 100% (noted as ****, high quality), with scores in between noted as ** or *** of mediocre quality. Additionally, all qualitative studies were assessed using the COREQ criteria and these scores were integrated in MMAT scores [24].

Primarily, data extraction was targeted on the effectiveness of leadership support and training programmes as a structural component of the integrated primary care implementation strategy on all possible outcomes e.g. individual or organizational. Secondarily, data were collected on the association between clinical leadership and integrated primary care with outcomes on the patient level and leadership skills needed for effective implementation of integrated primary care. We extracted additional data on study characteristics such as publication date, country, integrated-care setting, target patient population, design, data collection and participants and leadership perspective/approach.

We performed a narrative synthesis on results for leadership skills by categorizing outcomes using the Bell framework on collaboration [25]. This framework consists of five different themes: (1) shared ambition; (2) mutual gains; (3) relationship dynamics; (4) organization dynamics; and (5) process management [17]. After categorizing the data in these themes, we defined subthemes.

## Results

### Study characteristics

**Figure 2.**
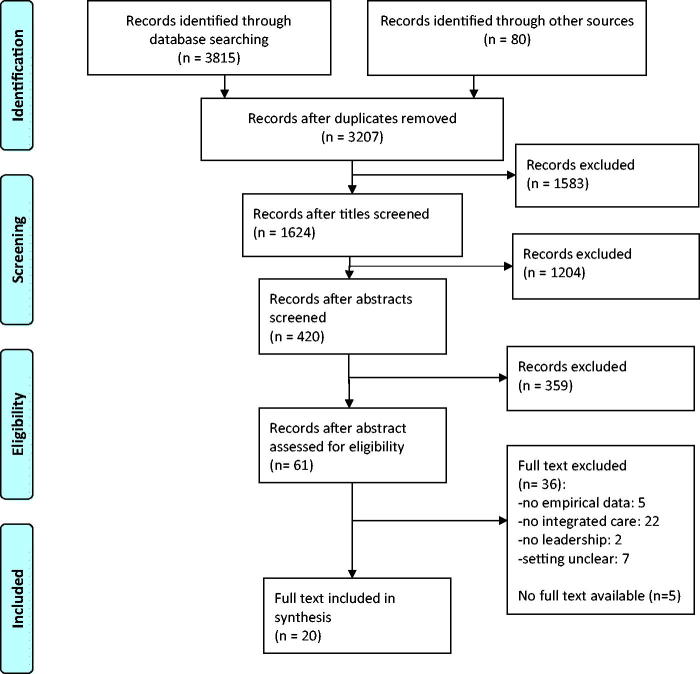
Diagram of information flow through phases of systematic review.

From the 3207 citations identified, 61 abstracts were found eligible of which 56 full-text articles were available (Figure 2). The researchers initially agreed on 48 articles for inclusion or exclusion (к = 0.86), on seven articles consensus was reached after discussion and for one article a third researcher was consulted. Finally, 20 articles were included (Table 1).

**Table 1.** Overall characteristics of the papers included in order reference by year of publication.

Reference, year, study quality	Country	Integrated-care setting (when specified target patient population)	Study design	Data collection	Participants
[26] 2006***	USA	Within primary care (depression care)	Qualitative	Telephone interview	5 community-based healthcare organizations/29 participating practices, 91 participants
[27]2006*	Canada	Between primary care and hospital (oncology care)	Qualitative	Longitudinal case study; non-participating observation of meetings, semi-structured interviews, documentary analysis	Local, regional and supra-regional multidisciplinary teams; five hospitals, 65 clinician leaders, medical and nursing staff members and managers
[28] 2007*	UK	Within primary care	Mixed methods, largely qualitative	Questionnaires, open-ended interviews, one-to-one consultations, discussion, individual case-study report, individual feedback and group presentations	6 district nurses/district nurse team leaders
[29] 2008**	Canada	Within primary care (palliative care)	Qualitative	Focus groups	8 primary care teams
[30] 2009**	Australia	Between primary care, hospital and residential (aged) care	Mixed methods, largely qualitative	Multi-method case-study: journals, interviews, focus groups and surveys	3 (student) nurse practitioners
[31] 2010***	Canada	Between primary care (addiction rehabilitation) and hospital (psychiatric)	Qualitative	Case study: interviews, focus groups, non-participant observation and document analysis	2 cases: 25 clinicians and administrators
[32] 2010**	France	Between primary care and hospital (community-dwelling elderly people with complex needs)	Qualitative	Interviews, observation, documents and focus groups	56 stakeholders: primary care, community-based services, hospitals and funding agencies
[33] 2010***	Canada	Within primary care	Qualitative	Exploratory case study and semi-structured interviews	14 family health teams
[34] 2012**	The Netherlands	Within primary care and between primary care and hospital (COPD, diabetes cardiovascular, psychiatric diseases)	Quantitative, cross-sectional design	Questionnaires:	22 disease-management partnerships
				Partnership synergy and functioning (PSAT)	
				Imp activeness disease-management partnership (ACIC)	218 professionals
[35] 2012**	UK	Within primary care (depression care)	Qualitative	Case study, in-depth interviews, documentary material	20 managers and practitioners
[36] 2013***	USA	Within primary care (diabetes, asthma)	Mixed methods	Qualitative: focus groups, clinical measures on diabetes and asthma and monthly practice implementation	Practice clinicians and managers of 76 practices; subsample of 12 practices for the focus group
				Quantitative: leadership and practice engagement scores rated by external practice coach	
[37] 2014**	USA	Between primary care and hospital	Mixed method, largely qualitative	Internal evaluation: Monthly performance data on three levels: beginner, middle and expert level on practice operation, clinical process and outcomes, and patient experience	9 collaborative practices involved, 260 000 patients, 450 professionals
				External evaluation: to determine how well the collaboration achieves aims	
[38] 2014**	USA	Within primary care	Mixed methods	Qualitative: interviews	22 practitioners from 5 pilots
				Quantitative: web-based survey	400 practitioners pilot and non-pilot
[39] 2014***	USA	Within primary care (depression care)	Mixed methods	Qualitative: site visits, observation, interviews, structured narratives	42 practices from 14 medical groups
				Quantitative: PHQ-9 scores, activation rates and remission rates of 1192 patients	
[40] 2015**	Australia	Within primary care (Aboriginals)	Qualitative	In-depth interview	5 senior leaders
[41] 2015****	Ireland	Within primary care	Qualitative	Semi-structured interview	2 primary care teams, 19 team members
[42] 2015**	USA	Within primary care (depression care)	Mixed methods	Qualitative: observation of quality improvement team monthly meetings	1 community health centre
				Quantitative: chart reviews	5044 adult patients
[43] 2015****	USA	Between primary care and hospital	Qualitative	Observation during site visits and interviews	9 sites, 80 participants from 12 professions
[44] 2017**	Japan	Within community and primary care (elderly)	Qualitative	Semi-structured interview and observation	26 medical professionals, including physicians, nurses, public health nurses, medical social workers and clerical personnel
[45] 2018***	The Netherlands	Within primary care (elderly)	Qualitative	Focus groups and observation	46 healthcare and social service professionals from four general practitioners practices

* = low quality, 25% on MMAT criteria.

** = mediocre quality, 50% on MMAT criteria.

*** = mediocre quality, 75% on MMAT criteria.

**** = high quality, 100% on MMAT criteria.

MMAT, Mixed methods appraisal tool; ACIC, assessment of chronic illness care; COPD, chronic obstructive pulmonary disease; PHQ-9**, **patient health questionnaire-9; PSAT, partnership self-assessment tool.

Studies included were conducted in Western countries, most in the USA (*n* = 7) and Canada (*n* = 4). The majority of studies used a qualitative design (*n* = 12) or a mixed methods design (*n* = 7). Two studies obtained the maximum MMAT scores (****); 16 studies were of mediocre and two of low quality. Studies were all conducted after 2006. In 12 studies, integrated care was targeted for specific chronic care diseases, e.g. depression and diabetes or the elderly population. Integrated-care interventions ranged from collaborative working and interprofessional collaboration to full Chronic Care Model implementation, including case management, and multidisciplinary teams and consortium building [28,31–34,36,38,44,45].

Ten studies explicitly mentioned the use of clinical leadership perspective [26–28,31,32,39,42–45]. Five studies focused on collective leadership [30,35,36,38,41]. Three articles mentioned that different leadership styles were needed in different phases of integrated-care implementation [27,32,39]. Five papers did not describe the leadership style addressed [29,30,33,38,41].

### Effectiveness of leadership interventions to improve integrated-care practice

We found no clinical trials on effectiveness of leadership interventions (support and preparation). Two studies, one mixed method study of mediocre quality [37] and one qualitative design of low quality [28], reported on the impact of a leadership intervention on integrated primary care practice. Bitton et al. investigated a leadership academy’s curriculum, including skill development and peer mentoring, that supported clinical leadership and change management [37]. Nineteen primary care practice teams, which consisted of clinical physician leaders, followed the leaderships academy’s curriculum during an 18-month period. The evaluation showed that clinical leadership behaviour improved (from 6.2 to 7.9, *P* <0.001, on the validated self-report patient centered medical home assessment, subscale ‘engaged clinical leadership;’ scores range from 0 (worst) to 12 (best)). Additional qualitative research findings suggested that leadership competencies must be augmented and learned at practice level to succeed in changing towards collaborative practice.

Alleyne et al. evaluated the clinical nursing leadership and action process model (CLINLAP), an approach to support firmly clinical (nursing) leadership [28]. This course included a two-day management-development workshop, group clinical supervision (90 min, weekly). Participants were additionally supported by a management development tool. In a qualitative evaluation, six district nurses stated that the CLINLAP model improved their capacity to enhance the quality of collaborative services provided to their patients, increased their confidence to perform and made implementing change more practical and manageable.

### Association between clinical leadership and integrated primary care practice and outcomes

Thirteen studies explored the association between leadership and integrated primary care (Table 2). Three studies used a quantitative, cross-sectional correlation design (MMAT **/***), and 10 studies used a qualitative design (MMAT * to ****). All these studies reported a positive influence of leadership on the integration of primary care and provided in-depth information on the most fruitful leadership approaches clinical leadership [27,31] and different types of collective leadership: team leadership and dispersed leadership [30,35,38,41]. Two studies revealed the value of continuity of leadership in person for implementation of integrated primary care [26,42]. Five studies reported explicitly that physician leaders were the most suited professionals for practicing the clinical leadership role [33,38,43–45]. One study found a strong relationship (β = 0.25) between effectiveness of leadership and chronic care model integrated partnership [34]. Two studies showed a significant correlation between strong leadership and patient outcome measures, such as patients’ activation (*r* = 0.6) and the proportion of patients having nephropathy screening (OR: 1.37) [36,39].

**Table 2.** Association between clinical leadership and integrated primary care and outcomes.

Reference	Study design	Leadership perspective	Integrated-care outcomes: Clinical measures or practice changes towards care integration: Teamwork, IPP, collaborative care
[26]	Qualitative	Clinical leadership	Leadership and durability of leadership was clearly associated with success in sustaining and spreading the intervention
[27]	Qualitative	Clinical leadership	Clinical leaders succeeded in influencing professional practices. However, it is obvious that change does not depend solely on the clinical leaders’ role
		Change leadership	
[30]	Mixed methods, largely qualitative	Clinical leadership	Collaboration and leadership attributes were interrelated and contributed to the impact of the emerging NP role. Leadership supported the work of the team
[31]	Qualitative	Clinical leadership	Clinical leadership had determinative positive influence on integration process
[33]	Qualitative	Clinical leader	Critical role of physician leadership in supporting collaborative care
		Change leadership	Essential role of a manager in supporting an sustaining collaborative care
[34]	Quantitative, cross-sectional	Overall leadership/senior leaders	Strong relationship (β = 0.25; *P* ≤ 0.01) between impact of disease management partnership (ACIC scores) and leadership (11 items on PSAT)
		Practice team leadership	
[35]	Qualitative	Leadership with focus on learning and knowledge management	Dispersed leadership approaches are the most appropriate for collaborative depression care
[36]	Mixed methods	Clinical leadership by practice leaders	Leadership was significantly associated with one clinical measure: the proportion of patients having nephropathy screening (OR: 1.37; 95%CI: 1.08–1.74)
			The odds of making practice changes were greater for practices with higher leadership scores at any given time (OR: 2.41–4.20). Leadership rated monthly on a 0–3 scale during one year
[38]	Mixed methods	Clinical leadership	Local physician leader facilitated sense of teamwork
[39]	Mixed methods	Top leadership	Statistically significant and moderately strong positive correlations for patient activation and strong leadership support (0.63)/strong care manager (0.62)/strong primary care practice champion (0.60)
		Primary care practice champion	
		Care manager	
[41]	Qualitative	Clinical leadership	Lack of leadership was considered to be a barrier to more efficient outcomes
			Formal leadership may not be fundamental to team working; team leadership would be advantageous
[42]	Mixed methods	Clinic QI leadership	Having onsite programme champions and durability of this leadership was important for implementation of collaborative care
[43]	Qualitative	Clinical leadership	IPP best practices emphasized role of physician leadership. Within historic hierarchy of medical care, physicians often are tone setting

ACIC, assessment of chronic illness care; OR, odds ratio; CI, confidence interval; IPP, interprofessional practice; NP, nurse practitioner; PSAT, partnership self-assessment tool.

### Leadership skills required for integrated primary care

Fourteen qualitative studies, one of high [43] and 13 of mediocre quality [26,29–33,35,36,38,40,42,44,45], described skills needed for integrated-care implementation and practice. Eleven studies reported skills related to relational dynamics such as encouraging team culture, facilitating interpersonal communication, fostering accountability and responsibilities of team members, positive role modelling and developing new professional roles [29,30,32,33,35,36,38,42–45]. Seven studies provided insight into organizational skills needed for clinical leaders: being visionary, decisive, being a catalyst and problem solving [26,30,31,36,40,43,45]. Process-management skills and change-management skills were reported in seven articles [26,29,31–33,36,45]. Two studies stated the need for leaders’ qualities to ensure the commitment of multidisciplinary team members to a shared purpose [32,35]. No skills required for Bell’s ‘mutual gains’ (understanding the various interests of the involved partners) category were mentioned (Table 3).

**Table 3.** Leadership skills required for integrated primary care.

Subthemes	Reference	Method for data collection	Leadership skills required
**Shared ambition (shared commitment of the involved partners)**			
Commitment	[32]	Interviews, observation, focus groups	Ensuring the broadening commitment of different health and social services
	[35]	In-depth interviews	Helping to develop and negotiate shared purpose
**Relationship dynamics (relational capital among the partners)**			
Team culture	[29]	Focus groups	Shared leadership: team members empowering each other in their team
	[30]	Case-study journals, interviews, focus group and surveys	Being able to function in a networked rather than a hierarchical manner
	[32]	Interviews, observation, focus groups	Maintain trusting relationships
			Establishing a collaborative culture: sensitivity to roles and contributions of different staff members
	[35]	In-depth interviews	Encouraging working in groups and teams
	[36]	Focus groups	Fostering culture of teamwork
			Sensitivity to issues learning to ‘work together’
	[43]	Observation during site visits, interviews	Valuing contribution of team member
			Creating safe space for team members
	[44]	Semi-structured interviews	Being able to consider the circumstances and ways of thinking of each discipline
Interpersonal communication	[29]	Focus groups	Conflict resolution
			Facilitate meetings
	[43]	Observation during site visits, interviews	Communicating expectations of team member overtly or implicitly
	[44]	Semi-structured interviews	Promoting the creation of good communication and close interaction between disciplines
Responsibilities	[29]	Focus groups	Foster accountability
			Divide responsibilities for different tasks to different team members
	[32]	Interviews, observation, focus groups	Clarifying dysfunctional areas and revising task distributions
	[42]	Observation of team monthly meetings	To champion protocol adherence
Role modelling	[30]	Case-study journals, interviews, focus group and surveys	Positive professional role modelling, to share expertise
			Developing transboundary role
	[33]	Semi-structured interviews	Positive physician role modelling
	[45]	Focus groups, observation	Taking initiative to build multidisciplinary teams
			Emphasizing the role of professionals close to patients, especially nurses and social workers
Role developing	[32]	Interviews, observation, focus groups	Refining and legitimating the role of the case manager
	[38]	Interviews, web-based survey	Providing confidence among individuals in adopting new roles
			Clarifying the scope of new role and responsibilities
			Providing a vehicle for incorporating new roles into routine practice
**Organization dynamics (governance arrangements among the partners)**			
Visionary	[26]	Telephone interviews	Visionary and committed
	[36]	Focus groups	Vision about the importance of the work
	[43]	Observation during site visits, interviews	Vision on IPP, including patient- and family-centred care, high-quality care
	[45]	Focus groups, observation	Passionate about delivering integrated, good quality, person-centred care
Decisiveness	[30]	Case-study journals, interviews, focus group and surveys	Evolving sense of authority
	[31]	Interviews, focus groups, non-participant observation and document analysis	Having determinative influence
			Having clearly decisiveness to implement practice changes
			Taking personal initiatives to set events in motion aimed at integrating healthcare resources
	[40]	In-depth interviews	Display of determination to persevere when faced with challenges an barriers to change
			Persistence in facing resistance to change from staff
	[45]	Focus groups and observation	Deciding on the composition of the multidisciplinary team
Catalyst problem solving	[36]	Focus groups	Serve as link between top management and staff
	[30]	Case-study journals, interviews, focus group and surveys	Taking positive action to resolve problems
	[40]	In-depth interviews	Overcome bureaucratic hurdles
**Process management (process steering among the partners)**			
Change management	[26]	Telephone interviews	Supporting improvement change culture, that permeates the organization
	[29]	Focus groups	Should have knowledge of change theory
	[32]	Interviews, observation, focus groups	Transforming the classic hierarchical relationship between GPs and nurses/case managers
	[33]	Semi-structured interviews	Should encourage change
			Should be innovative, creative and possess project development and management skills
	[36]	Focus groups	Test and implement innovations
Project management	[29]	Focus groups	Public speaking, presentation skills, coaching skills, writing proposals and abstracts
	[31]	Interviews, focus groups, non-participant observation and document analysis	To empower individuals to participate in transformation activities
	[32]	Interviews, observation, focus groups	Tailoring to the various phases of the diagnostic, design and implementation process
	[36]	Focus groups	Taking personal initiative to set events in motion aimed at integrating healthcare resources
	[45]	Focus groups, observation	Networking at the strategic level: connecting primary and secondary care, social services, and the community

GP, general practitioner; IPP, interprofessional practice; QI, quality improvement.

Bells Framework consists of [1] shared ambition, [2] mutual gains, [3] relationship dynamics, [4] organization dynamics and [5] process management.

Mutual gains was not mentioned.

## Discussion

### Main findings

In this systematic review, we found no controlled studies on the effectiveness of clinical leadership on integrated primary care practice and outcomes on patient level. Two articles suggested that leadership support programmes may contribute to preparing leaders for the implementation of integrated primary care. Leaders’ relational and organizational skills as well as process-management and change-management skills were considered important to improve care integration but were never tested. Physicians were appointed as the most adequate leaders. Most empirical studies included in the review were explorative by nature and of mediocre quality. The focus on leadership as a research target in relation to integrated care seems to be a new phenomenon as all studies selected were conducted after 2006.

### Strengths and limitations

The main strength of this first systematic review covering the association between leadership and integrated primary care is that we performed a sensitive search with few limitations. However, we may still have missed potentially relevant articles because the underlying concepts of integrated care as well as leadership are not yet clearly defined. This also might have given rise to multiple interpretations during the selection process. To overcome this problem, the screening process was carried out by two researchers with at least ten years of experience in the field of integrated primary care. Moreover, they independently screened 420 abstracts and 56 full-text articles, with a high agreement rate.

Another limitation is that our search was limited to databases of clinical research when studying a management topic. Since this review focused on clinical leadership, we argue that we are probably able to identify the most relevant papers in the databases used. We tried to diminish this factor further by using snowball methods and manual searching of key articles on the implementation of integrated care including studies published in organizational science journals.

### Comparison with existing literature

*Effectiveness of leadership interventions.* This review revealed that the use of leadership as the implementation strategy, although recommended in the Chronic Care Model and by many experts in the field, was hardly applied or described since we only found two studies of low and mediocre quality that evaluated leadership-training interventions aimed at structurally supporting implementation processes of integrated care. This shows that the importance of leadership to integrated primary care does not yet transcend the level of opinions.

*Association between clinical leadership and integrated primary care.* The association between leadership and integrated care is not substantiated with firm evidence [20]. This review appoints physicians as the professionals most capable of transforming care towards more integration. Until now, physicians have indeed been the principal players in either opposing or supporting successful transformative efforts [46]. Recognition of the need for physicians’ leadership role development and support and increased attention on clinicians’ collaboration and leadership skills were recently stipulated in physicians competency profiles (i.e. CANMED roles) [12,47]. Other professionals, e.g. nurses and social workers, may lack the hierarchical position in comparison with physicians and possibly need more support to perform their leadership role; skills to perform this role are not automatically present in professionals and the importance of supporting professionals in their leadership role is still underestimated [20].

*Required leadership skills.* Our review indicates that some relational leadership styles, especially collective leadership and team leadership, may be fruitful in the implementation of integrated primary care. Relational and organizational skills, as well as process-management and change-management skills, such as communicating expectations, maintaining trusting relationships and creating safe space, were also found important in other reviews [8,20]. Remarkably, the need for leaders to be able to understand mutual gains was not mentioned in the papers included. A possible explanation is that the ability to oversee the consequences of care integration for the organizations involved is complicated, as competitive dynamics may hinder crossing organizational borders [48].

### Implications for research and/or practice

This review underlines the need for innovation in leadership research, training and practice. Furthermore, it shows that evaluating leadership in integrated primary care is challenging. Future research could benefit from better-defined concepts and a clear research agenda on leadership in the context of integrated primary care [20]. Leadership skills identified in this review can fuel the development of leadership programmes in vocational training curricula and interprofessional education. Evaluation of complex educational leadership interventions and the complex integrated primary care setting may ask for innovative research designs instead of classical randomized controlled trials. An example of such an innovative design is the longitudinal mixed methods case study to evaluate DementiaNet, an implementation programme for networked primary dementia care [49]. This design enabled a better understanding of the effects and working mechanisms. Outcomes in this study were network maturity and quality of care. These outcomes and their interrelatedness, combined with leadership skills assessment, are also relevant for the evaluation of clinical leadership programmes in the integrated primary care setting.

## Conclusion

In the field of primary care, experts consider leadership to be a relevant factor for good-quality integrated care. However, this review revealed that there is no firm evidence for its positive impact. The evidence available is limited mainly to qualitative studies. Leadership support aimed at developing skills for integrated-care implementation is probably effective but a more profound evidence base is required. We therefore, advocate the development of higher-quality knowledge about leadership focused on the implementation of the integrated-care practice.

## Supplementary Material

Search strategy Pubmed

PRISMA 2009 Checklist
