# Supplementary material for: Clinical leadership and integrated primary care: A systematic literature review
Source: Eur J Gen Pract. 2018 Nov 26;25(1):7–18. doi: 10.1080/13814788.2018.1515907 (PMC6394325; doi:10.1080/13814788.2018.1515907)
Supplement: Search strategy Pubmed [file IGEN_A_1515907_SM4053.pdf]

Search strategy Pubmed (final)
Search on November 1st 2015; Alert until June 30th 2018

Introduction:
The research questions of this systematic review: to explore: (1) effectiveness of programmes to support leadership for integrated primary care (2) the relationship between leadership and integrated primary care and (3) important leadership skills for integrated primary care.

Search words:

LEADERSHIP
"leadership"[Mesh] OR
leader*[tiab] OR
champion[tiab] OR
champions[tiab] OR
coordinator[tiab] OR
clinical governance[tiab] OR
entrepreneurship[tiab] OR
seniority[tiab]

AND

CHRONIC CARE MODEL
"Chronic Disease"[Mesh] AND ("Managed Care Programs"[Mesh] OR "Delivery of Health Care"[Mesh]) OR
chronic care model*[tiab] OR
ccm[tiab] OR
ccms[tiab]

LEADERSHIP

AND

INTEGRATED CARE

Delivery of Health Care, Integrated"[Mesh] OR
Integrated care[tiab] OR
Integrating care[tiab] OR
Integrated healthcare[tiab] OR
Integrating healthcare[tiab] OR
Integration of care[tiab] OR
coordinated care[tiab] OR
coordinating care[tiab] OR
coordinated healthcare[tiab] OR
coordinating healthcare[tiab] OR
coordination of care[tiab] OR
coordination of healthcare[tiab] OR
Integrated health service*[tiab] OR
Integrating health service*[tiab] OR
Integrated healthservice*[tiab] OR
Integrating healthservice*[tiab] OR
Integration of health services[tiab] OR
Integration of healthservices[tiab]) OR
Collaborative Care[tiab] OR

Collaborative Health care[tiab] OR
Collaborative Healthcare[tiab] OR
Collaborative Service*[tiab] OR
Collaborative Healthservice*[tiab] OR
Collaborative Health service[tiab] OR
Interprofessional collaboration[tiab] OR
Interprofessional cooperation[tiab] OR
Interprofessional work[tiab] OR
Inter-professional collaboration[tiab] OR
Inter-professional cooperation[tiab] OR
Inter-professional work[tiab] OR
Interorganisational collaboration[tiab] OR
Interorganisational cooperation[tiab] OR
Inter-organisational collaboration[tiab] OR
Inter-organisational cooperation[tiab] OR
Interorganizational collaboration[tiab] OR
Interorganizational cooperation[tiab] OR
Inter-organizational collaboration[tiab] OR
Inter-organizational cooperation[tiab] OR
Care coordination[tiab])

Explanatory memorandum:
The starting point of our search was the question: what helps health care professionals involved with integrated care in their leading role. We soon discovered however that only few studies were executed that could help us answer this question directly. Therefore we decided to add the two secondary questions and we searched for studies on the association between leadership and integrated primary care with outcomes on the patient level as well as leadership skills needed for effective implementation of integrated primary care.
